# Effects of concurrent intravenous morphine sulfate and naltrexone hydrochloride on end-tidal carbon dioxide

**DOI:** 10.1186/1477-7517-9-13

**Published:** 2012-03-15

**Authors:** Veeraindar Goli, Lynn R Webster, Michael J Lamson, Jody M Cleveland, Kenneth W Sommerville, Eric Carter

**Affiliations:** 1 Pfizer Inc, Cary, NC, USA; 2 Duke University Medical Center, Durham, NC, USA; 3 Lifetree Clinical Research, Salt Lake City, UT, USA; 4 Allergan, Inc. (formerly Pfizer Inc), Irvine, CA, USA; 5 Pfizer Inc, 4000 CentreGreen Way, Suite 300, Cary, NC 27513, USA

**Keywords:** Morphine, Naltrexone, Opioid, Opioid antagonist, Respiratory depression, Opioid overdose, Drug abuse

## Abstract

**Background:**

Respiratory depression, a potentially fatal side-effect of opioid-overdose, may be reversed by timely administration of an opioid antagonist, such as naloxone or naltrexone. Tampering with a formulation of morphine sulfate and sequestered naltrexone hydrochloride extended release capsules (MS-sNT) releases both the opioid morphine and the antagonist naltrexone. A study in recreational opioid-users indicated that morphine and naltrexone injected in the 25:1 ratio (duplicating the ratio of the formulation) found MS-sNT reduced morphine-induced euphoric effects vs intravenous (IV) morphine alone. In the same study, the effects of morphine + naltrexone on end-tidal carbon dioxide (EtCO_2_), a measure of respiratory-depression, were evaluated and these data are reported here.

**Methods:**

Single-center, placebo-controlled, double-blind crossover study. Non-dependent male opioid users were randomized to receive single IV doses of placebo, 30 mg morphine alone, and 30 mg morphine + 1.2 mg naltrexone. EtCO_2 _was measured by noninvasive capnography.

**Results:**

Significant differences in EtCO_2 _least-squares means across all treatments for maximal effect (E_max_) and area under the effect curve (AUE_0-2_, AUE_0-8_, AUE_0-24_) were detected (all p ≤ 0.0011). EtCO_2 _E_max _values for morphine + naltrexone were significantly reduced vs morphine alone (42.9 mm Hg vs 47.1 mm Hg, p < 0.0001) and were not significantly different vs placebo (41.9 mm Hg). Median time to reach maximal effect (TE_max_) was delayed for morphine + naltrexone vs morphine alone (5.0 h vs 1.0 h).

**Conclusions:**

Results provide preliminary evidence that the naltrexone:morphine ratio within MS-sNT is sufficient to significantly reduce EtCO_2 _when administered intravenously to non-dependent male recreational opioid-users. Further studies with multiple measures of respiratory-function are warranted to determine if risk of respiratory depression is also reduced by naltrexone in the tampered formulation.

## Background

In the United States in 2007, more than one-third (36%) of all poisoning deaths involved opioid analgesics (drugs usually prescribed to relieve pain) [1]. Since 1999, poisoning deaths in the United States involving opioid analgesics have more than tripled [1,2]; deaths from opioid analgesics have surpassed those from heroin and cocaine [3,4].

Although prescription opioids may be formulated for oral use, they are often taken intravenously or intranasally when abused [5,6]. As tolerance to opioid psychoactive effects increases with use over time, the user often progresses from the oral route to the intranasal or intravenous (IV) route to attain greater opioid effects and more rapid onset of action [5,7-10].

Respiratory depression is the leading cause of death following opioid overdose [11]. Opioids interact with μ-opioid receptors, reducing the central nervous system responsiveness to hypercapnia and hypoxia, and the peripheral chemoreceptor response to hypercarbia [11-14]. Respiratory depression involves inadequate response to hypercapnia or hypoxia, resulting in a decrease in respiratory rate and/or decrease in minute ventilation below normal [15,16]. Respiratory depression can be defined as a deviation of respiratory rate, pulse oximetry value, or carbon dioxide tension from an arbitrary threshold [17,18]. Opioid-induced respiratory depression can occur within minutes of IV injection and typically can result in death in < 1 to 3 hours following opioid exposure [7,19,20].

Respiratory depression can be reversed by timely administration of an opioid antagonist [21]. However, as respiratory depression can develop within minutes, the interval for successful intervention can be short [7,19,22]. In addition, timely intervention is not always possible because overdose often occurs in a non-hospital setting [23]. In a review of medical examiner data, more than half of the individuals who died from accidental drug overdoses had already expired before they reached the emergency department [24].

Naltrexone, a competitive, selective μ-opioid receptor antagonist, is available in oral and injectable formulations for treatment of alcohol dependence and blockade of opioid effects [25-27]. Although it is not currently indicated for reversal of opioid-induced respiratory depression, naltrexone may provide an early intervention option to reduce deaths from opioid-induced respiratory depression.

Naltrexone is a component of morphine sulfate and naltrexone hydrochloride extended release capsules (MS-sNT, EMBEDA^® ^), indicated for moderate-to-severe pain when a continuous, around-the-clock opioid analgesic is needed for an extended period of time. The formulation contains pellets of extended release morphine, each with a sequestered core of naltrexone hydrochloride at a fixed 25:1 ratio of morphine sulfate:naltrexone hydrochloride (4% naltrexone) [28,29]. Taken as directed, morphine provides analgesia while the naltrexone remains sequestered [29-32]. The product is designed so that tampering by crushing will rapidly release morphine and naltrexone. The released naltrexone is available to reduce morphine-induced effects, such as euphoria [28,29]. The quantity of naltrexone sequestered in MS-sNT (e.g., 4% of the morphine dose or 1.2 mg of naltrexone in a capsule containing 30 mg of morphine) is substantially lower than that used clinically (50 mg once daily oral dose) for blockade of opioid effects or alcohol dependence or that which yields 90% occupancy of human opiate receptors (32-48 mg total daily oral dose) [26,28,33]. Results of an earlier study in non-dependent opioid-experienced volunteers have shown that the naltrexone in crushed MS-sNT taken orally was successful in mitigating morphine-induced subjective effects, such as drug liking and euphoria [29]. Similarly, this study in non-dependent recreational opioid users was designed to simulate IV injection of tampered MS-sNT and was conducted by injecting morphine sulfate (30 mg) and naltrexone hydrochloride (1.2 mg) in the same 25:1 ratio in the MS-sNT formulation. When both morphine and naltrexone were administered intravenously, naltrexone reduced morphine-induced euphoric effects [34]. This study also included measurements of end-tidal carbon dioxide (EtCO_2_) concentrations using noninvasive capnography as an exploratory end point. As EtCO_2 _monitoring in the operating room is standard practice for evaluating ventilation [35], it may be considered a surrogate end point of respiratory depression [36]. The EtCO_2 _measurements from this IV morphine and naltrexone study [34] are presented here to consider whether the naltrexone within MS-sNT could attenuate respiratory depression in opioid overdose if MS-sNT were crushed and taken intravenously.

## Methods

### Study design

The study was a single-center, placebo-controlled, randomized, double-blind, 3-way crossover study [34]. The primary objective of the study was to determine drug liking and euphoric effects of IV morphine alone and IV morphine + IV naltrexone. These data were published previously [34]. Evaluation of effects on respiratory depression as measured by EtCO_2 _was a secondary, exploratory objective of the study [34] and these data are reported here. The study was conducted in accordance with current US Food and Drug Administration regulations, International Conference on Harmonisation Guidelines, Good Clinical Practice standards, Declaration of Helsinki, and local ethical and legal requirements. Subjects signed an informed consent form approved by an institutional review board (Compass IRB, Mesa, AZ) before participation in the study [34,37,38].

### Subjects

Subjects were enrolled if they were male between the ages of 18 and 50 years and were opioid-preferring, non-opioid-dependent, recreational drug users who reported inappropriately taking a prescription opioid to achieve a high at least 5 times within the previous 12 months [34]. Subjects were to be in generally good health as determined by medical history and physical examination. Subjects with a history of significant pulmonary, neurological, hepatic, renal, endocrine, cardiovascular, gastrointestinal, or metabolic disease were excluded [34].

### Interventions

Investigators determined whether subjects were opioid-dependent by administration of an IV challenge of 0.4 mg naloxone [34]. Subjects identified as non-dependent were randomized to receive either 10 mg morphine or placebo IV separated by a 1-day washout to determine ability to discriminate morphine from placebo based on responses to the Drug Effects Questionnaire (DEQ), and the Cole/Addiction Research Center Inventory (ARCI) Stimulation-Euphoria modified scale [34,39]. All subjects who were able to discriminate morphine from placebo entered the double-blind treatment phase during which subjects received each of three study treatments in a random order (separated by a 6-day outpatient washout period): 1) a single 30 mg IV bolus of morphine immediately followed by a single IV bolus of naltrexone placebo; 2) a single 30 mg IV bolus of morphine immediately followed by a single 1.2 mg IV bolus of naltrexone; or 3) a single IV bolus of morphine placebo immediately followed by a single IV bolus of naltrexone placebo. Each IV administration was spaced < 30 seconds apart [34]. The subjects were required to have a negative screen for drugs and alcohol on readmission for each treatment period.

### EtCO_2 _measures

Assessments were made immediately before dosing (t = 0) and immediately following pharmacokinetic sampling at 5, 15, 30, 45, 60, 120, 150, 180, 210, 240, 270, 300, 360, 480, 720, and 1440 minutes post-dose. EtCO_2 _was measured in millimeters of mercury (mm Hg) using noninvasive capnography [40]. A CAPNOGARD^® ^EtCO_2 _monitoring system (Novametrix Medical Systems, Inc., Wallingford, CT, USA) was used with an airway adapter and subject mouthpiece. Subjects were instructed to breathe normally through the mouthpiece for 1 minute. For each respiratory assessment, maximum effect (E_max_), time to maximum effect (TE_max_), and area under the effect curve computed using the linear trapezoidal rule (AUE, from time 0 to 2, 8, and 24 hours post-dose [AUE_0-2_, AUE_0-8_, and AUE_0-24_]) were summarized. Clinical significance was not assessed in this study.

### Statistics

A linear mixed model (PROC Mixed SAS^® ^), with fixed effects for sequence, period, and treatment and a random effect for subject nested in sequence, was used to compare EtCO_2 _measures among treatments [34]. Least squares (LS) means and 95% confidence intervals (CIs) were determined for each treatment as well as the LS mean differences and 95% CIs for each treatment comparison. For E_max_, AUE_0-2_, AUE_0-8 _and AUE _0-24_, pairwise comparisons of each treatment were performed. Analysis populations were:

• Safety population: all subjects who received at least 1 dose of study drug during the double-blind treatment phase.

• PD population: all subjects who received at least 1 dose of study drug during the double-blind treatment phase and provided at least 1 PD assessment during the double-blind treatment phase.

• Evaluable PD population: all subjects in the PD population who completed the morphine + naltrexone treatment and at least 1 other study treatment during the double-blind treatment phase.

## Results

Of the 28 male subjects in the Safety population who received at least 1 dose of study drug, 2 discontinued; 1 for noncompliance and 1 because of a tooth infection. All 28 subjects were included in the PD population. Subjects in the Safety population had a mean age of 23.8 years (range, 18-36 years) and 89% were white. All subjects had a history of recreational drug abuse as a criterion of study entry and many were current smokers (nicotine) and regularly consumed alcohol. Of the 2 subjects who discontinued, 1 did so during placebo treatment and prior to the morphine + naltrexone treatment (i.e., only completed morphine), the other during morphine treatment and prior to placebo (i.e., only completed morphine + naltrexone). The Evaluable PD population, therefore, included 27 subjects for morphine, 27 for morphine + naltrexone, and 26 for placebo.

Primary and secondary outcomes have been reported previously [34] and are briefly summarized here. Morphine + naltrexone significantly reduced mean pharmacodynamic assessments of high, euphoria and drug liking compared with morphine alone, although both active treatments were significantly higher than placebo [34]. Mean pupil diameter was significantly reduced with morphine compared with placebo and morphine + naltrexone [34].

**Figure 1:**
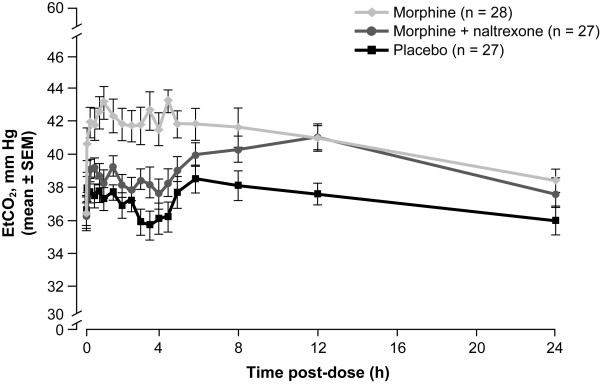
**Mean EtCO_2 _(mm Hg) ± SEM over 24 hours post-dose (PD population)**.

**Figure 2:**
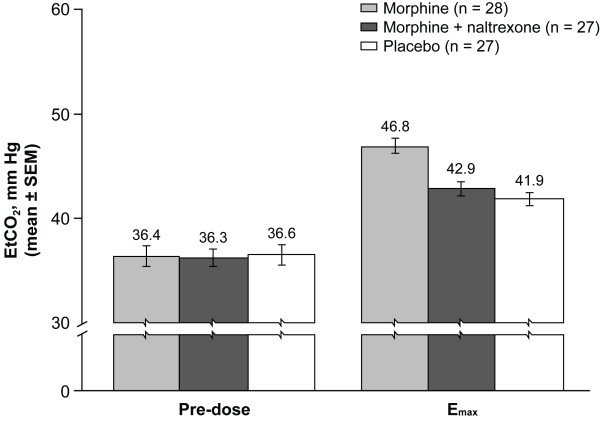
**Mean pre-dose EtCO_2 _and post-dose E_max _EtCO_2 _(mm Hg)(PD population)**. Error bars represent SEMs.

Mean EtCO_2 _values over time are illustrated in Figure 1. Morphine administered alone increased mean EtCO_2 _vs placebo over the entire 24-hour post-dose period, with increases apparent as early as 5 minutes post-dose. The EtCO_2 _profile over time for morphine + naltrexone remained significantly below that of morphine alone for approximately 6 hours, and numerically lower through 12 hours (Figure 1). The increase from pre-dose EtCO_2 _values to E_max _for morphine + naltrexone (mean, 6.6 mm Hg; median, 6.0 mm Hg) and placebo (mean, 5.3 mm Hg; median 5.0 mm Hg) were smaller than for morphine alone (mean, 10.4 mm Hg; median, 9.0 mm Hg) (Figure 2).

Individual EtCO_2 _values ranged from 20 to 46 mm Hg prior to dosing. During the double-blind treatment phase, individual EtCO_2 _E_max _values ranged from 39 to 61 mm Hg for morphine alone; 38 to 49 mm Hg for morphine + naltrexone; and 37 to 49 mm Hg for placebo. Individual EtCO_2 _values for the morphine + naltrexone treatment never exceeded the maximum value for placebo. The range of individual changes from pre-dose EtCO_2 _to E_max _for all 3 treatments was similar (morphine, 5.0 to 21.0 mm Hg; morphine + naltrexone, 1.0 to 15.0 mm Hg; placebo, 0.0 to 19.0 mm Hg).

In the Evaluable PD population, statistically significant differences were observed for EtCO_2 _LS means across all treatments for E_max_, AUE_0-2_, and AUE_0-8 _(all p < 0.0001) and AUE_0-24 _(p = 0.0011) (Table 1). Treatment comparison for EtCO_2 _E_max _indicated that for morphine + naltrexone, E_max _was significantly reduced vs E_max _for IV morphine alone (p < 0.0001), while the E_max _values for morphine + naltrexone vs placebo were not significantly different (p = 0.3064). There was no difference between morphine + naltrexone and morphine alone in EtCO_2 _AUE_0-24 _(p = 0.3256). Median TE_max _was delayed for morphine + naltrexone vs morphine alone (5.0 hours vs 1.0 hour) and placebo (2.0 hours). The mean respiratory rates showed a slight suppression for the morphine group at all time points after dosing (maximum mean reduction from pre-dose 1.71/min) but no group showed a mean lower than 16/min.

**Table 1:** Summary assessment of EtCO_2 _(Evaluable PD Population)

Parameter*	Morphine N = 27	Morphine + Naltrexone N = 27	Placebo N = 26
TE_max_, h, median (range)	1.0 (0.3, 8.0)	5.0 (0.1, 12.0)	2.0 (0.0, 24.0)

E_max_, mm Hg	47.1 (45.7, 48.5)^a^	42.9 (41.5, 44.3)^b^	41.9 (40.5, 43.3)

AUE_0-2 _(h•mm Hg)	11.1 (8.8, 13.5)^a^	4.8 (2.4, 7.1)^a,b^	1.6 (-0.8, 4.0)

AUE_0-8 _(h•mm Hg)	42.7 (33.0, 52.3)^a^	21.1 (11.5, 30.8)^a,b^	4.6 (-5.2, 14.5)

AUE_0-24 _(h•mm Hg)	96.3 (63.3, 129.3)^a^	74.6 (41.6, 107.6)^a^	11.8 (-21.9, 45.4)

*Except for TE _max _all values are LS means (95% CI); TE _max _is median (range)

Comparisons vs placebo: ^a^p < 0.05

Comparisons vs morphine: ^b^p < 0.05

AUE = area under the effect curve; CI = confidence interval; E_max _= maximum effect; LS = least squares; NS = not significant; TE_max _= time to maximum effect

## Discussion

With the increase in opioid-related drug overdose deaths in the United States comes a need for harm reduction strategies not only to address the issues of misuse, abuse, and diversion at their sources, but also to consider what might occur in the community setting. The naltrexone contained in MS-sNT was designed for release if there is tampering, to mitigate the psychogenic effects of morphine, such as drug liking, high, and euphoria. The current analysis reports results of an exploratory outcome (which was part of the Webster et al. study [34]), which assessed the impact of naltrexone on EtCO_2_, a surrogate measure of respiratory depression, when naltrexone was coadministered intravenously with morphine in the ratio (4%) present in the MS-sNT formulation. It was intended as a clinical simulation of the effects if MS-sNT were to be completely crushed, solubilized, and injected by non-dependent, recreational opioid users.

In this study, no subject receiving any treatment was reported by the investigator to have clinically relevant respiratory depression at the IV morphine dose administered (30 mg). However, compared with placebo, morphine alone resulted in immediate increases in EtCO_2 _that were maintained for 24 hours post-dose. Morphine + naltrexone (25:1 ratio) significantly reduced maximum EtCO_2 _vs morphine alone. No significant difference was detected between the combination morphine + naltrexone and placebo in EtCO_2 _levels at E_max_, emphasizing the PD effect of morphine displacement from the μ-opioid receptor by naltrexone.

There is no consensus on which measures define clinically relevant respiratory depression, since it can vary by patient depending on factors such as their condition, position, sleep state, etc. For this study, EtCO_2 _at E_max _was considered a sensitive early measure of respiratory depression because it is an expression of the acute effect of the opioid at the moment. A rising EtCO_2 _can reflect hypoventilation, which may precede other signs of respiratory depression such as hypoxia [36]. Although there is no consensus on measures that define clinically relevant respiratory depression, an EtCO_2 _> 50 mm Hg has been used as a criterion for respiratory depression in clinical trials during procedural sedation [36,41,42]. In the current trial, the highest individual levels measured with the morphine + naltrexone and the placebo treatments were 49 mm Hg. A retrospective review of the data indicates that there were 4 subjects who had values > 50 mm Hg, all with the morphine treatment. The highest individual level with the morphine treatment was 61 mm Hg. A change in EtCO_2 _of ≥ 10 mm Hg had been suggested as an additional indicator of respiratory depression in patients under sedation [36]. Three subjects in the placebo arm, 6 in the morphine + naltrexone arm, and 13 in the morphine arm (including 3 of the 4 subjects with EtCO_2 _≥ 50 mm Hg), had changes ≥ 10 mm Hg. As subjects in this study were awake rather than sedated, the clinical relevance of these changes is unknown, although the changes of increasing EtCO_2 _may indicate early respiratory suppression.

Results of the study reported here for EtCO_2 _are similar to those reported by Stoops et al [7], in which physiological measures for morphine (arterial oxygen saturation and pupil diameter) were apparent within 5 minutes of IV drug administration (5, 10, and 20 mg morphine doses) and were maintained for approximately 6 hours following opioid dosing [7]. The subjective psychodynamic effects of IV administration of opioids are also apparent early, within 10 minutes, of opioid dosing, but begin to decrease within 30 minutes after dosing [7,34]. The observation that the physiological effects (EtCO_2_, oxygen saturation and pupil diameter) last much longer than the subjective effects has clinical relevance. Individuals who abuse opioids may be unaware of the continuing physiological effects of the first opioid dose and the potential for compounding effects on respiratory depression by injecting additional opioid, and may re-inject opioid once subjective effects have begun to subside [7]. This also illustrates that μ-opioid receptor PD effects have different rates of onset, duration of effects, and slopes of dissipation. The effect of naltrexone coadministered with morphine on reduction of EtCO_2 _values vs morphine alone was evident as early as 5 minutes and continued through 8 hours when the difference diminished (Figure 1). The prolonged action of morphine was longer than the effect of the naltrexone blockage, as the mean EtCO_2 _values were similar between morphine + naltrexone compared with morphine alone at 12 hours (see Figure 1). This suggests that additional dosing of antagonist may be needed if a patient were to have clinically significant morphine-induced respiratory depression.

Results of this exploratory outcome are limited to 1 surrogate measure of respiratory function, using a set dose of morphine/naltrexone, at designated time frames, in a small, male population in a specific setting. The effects of naltrexone alone were also not evaluated in this study. Clinical significance was not assessed in this study, although no subjects were reported by the investigator to show signs of clinically relevant respiratory depression. In addition, this study was a clinical simulation of IV administration of tampered (fully crushed) MS-sNT, rather than administration of actual tampered product, due to concern about potential injury to the cardiopulmonary system from injection of excipients such as talc and particulate matter that may be present in crushed MS-sNT. This further limits the extrapolation to effects on EtCO_2 _of abuse by injection of actual crushed MS-sNT. Future studies using an array of validated and sensitive measures of respiratory depression, at opioid doses relevant for respiratory depression, would be needed to demonstrate consistent changes in these parameters and to document effects on clearly defined respiratory function. Assessments to accurately measure the early onset of opioid-induced respiratory depression and withdrawal would provide valuable clinically meaningful information. However, while these assessments can be performed under research conditions in a clinical setting, it is important to recognize that overdoses often occur in the community setting with many confounding factors and without access to measurement tools.

## Conclusions

Results of this exploratory analysis demonstrate significant reduction in EtCO_2 _after IV morphine + naltrexone vs morphine alone. Further, EtCO_2 _levels were not significantly different between morphine + naltrexone and placebo, emphasizing the PD effect of naltrexone displacement of morphine from the μ-opioid receptor. This is the first indication that abuse of MS-sNT by crushing and injection may not only abate morphine-induced drug liking and euphoria but may also attenuate morphine-induced effects on EtCO_2_. Further studies using an array of sensitive measures of respiratory depression to determine the impact of coadministration of morphine sulfate and naltrexone hydrochloride in the 25:1 ratio present in MS-sNT on measures of respiratory depression may increase understanding of the harm reduction potential of MS-sNT.

## Disclosures

LRW has received consultant and advisory board honoraria and research funding from Adolor Corp.; Alkermes, Inc.; Allergan, Inc.; American Board of Pain Medicine; Astellas Pharma US, Inc.; AstraZeneca; Bayer HealthCare; BioDelivery Systems International; Boston Scientific, Corp.; Cephalon, Inc.; Collegium Pharmaceuticals; Covidien; Covidien Mallinckrodt; Eisai Co., Ltd.; Elan Pharmaceuticals; Gilead Sciences; GlaxoSmithKline; Janssen Pharmaceuticals, Inc.; Meagan Medical; Medtronic; Nektar Therapeutics; NeurogesX, Inc; Nevro Corporation; PharmacoFore, Inc.; Purdue Pharma; Shionogi USA, Inc; St Renatus; SuCampo Pharma Americas, USA; TEVA Pharmaceuticals (Sub-I); Theravance, Inc.; Vertex Pharmaceuticals; Xanodyne Pharmaceuticals, Inc.; and King Pharmaceuticals^®^, LLC which was acquired by Pfizer in March 2011. VG, MJL, and KWS are all employees of Pfizer Inc. EC is currently an employee of Allergan, Inc., and was formerly an employee of Pfizer Inc. JMC is currently an employee of United Therapeutics Corporation, and was formerly an employee of Pfizer Inc.

## Competing interests

This study was sponsored by Alpharma Pharmaceuticals, LLC, which was acquired by King Pharmaceuticals^®^, Inc, which was acquired by Pfizer Inc in March 2011.

## Authors' contributions

LRW was a principal study investigator, contributed to the conceptualization, design, conduct and analyses of the study, interpretation, and writing of this manuscript. VG, MJL, JMC, KWS, and EC contributed to the conceptualization, design, interpretation, and writing of the manuscript. All authors read and approved the final manuscript.
